# Supplementary material for: Nondestructive Detection of Polyphenol Oxidase Activity in Various Plum Cultivars Using Machine Learning and Vis/NIR Spectroscopy
Source: Foods. 2025 Dec 13;14(24):4297. doi: 10.3390/foods14244297 (PMC12732983; doi:10.3390/foods14244297)
Supplement: Supplementary file 1 [file foods-14-04297-s001.zip › foods-4013729-supplementary.pdf]

Table S1. Results of SVM-R modeling of Khormaei cultivars with various spectral preprocessings

Kernel	Preprocessing	Training			Validation			Test		
		R^2	RMSE	RPD	R^2	RMSE	RPD	R^2	RMSE	RPD
Linear	No Preprocessing	0.98	0.0003	6.91	0.79	0.00086	2.25	0.16	0.00209	1.12
	SNV	0.98	0.00029	7.04	0.15	0.00201	0.96	0.14	0.00211	1.11
	MSC	0.98	0.00029	7.01	0.2	0.00205	0.94	0.24	0.00198	1.19
	Normalization	0.98	0.0003	6.86	0.81	0.00081	2.38	0.25	0.00197	1.19
	Moving Average	0.98	0.0003	6.87	0.78	0.00088	2.18	0.17	0.00207	1.13
	Gaussian Filter	0.98	0.0003	6.87	0.76	0.00091	2.12	0.17	0.00207	1.14
	Median Filter	0.98	0.0003	6.87	0.81	0.00082	2.35	0.1	0.00215	1.09
	Detrend	0.98	0.00029	6.99	0.09	0.00179	1.08	0.05	0.00233	1.01
	Mean Centering	0.98	0.0003	6.9	0.79	0.00086	2.25	0.17	0.00207	1.13
RBF	No Preprocessing	0.88	0.0007	2.92	0.57	0.00123	1.58	0.57	0.00149	1.57
	SNV	0.7	0.00112	1.83	0.14	0.00174	1.11	0.06	0.00234	1
	MSC	0.71	0.0011	1.88	0.16	0.00172	1.13	0.08	0.00217	1.08
	Normalization	0.89	0.00067	3.05	0.56	0.00124	1.55	0.57	0.0015	1.57
	Moving Average	0.88	0.0007	2.93	0.57	0.00122	1.58	0.57	0.00149	1.58
	Gaussian Filter	0.87	0.00074	2.76	0.54	0.00127	1.52	0.52	0.00157	1.5
	**Median Filter**	**0.91**	**0.00062**	**3.32**	**0.62**	**0.00115**	**1.69**	**0.63**	**0.00138**	**1.7**
	Detrend	0.57	0.00133	1.55	0.18	0.00203	0.95	0.11	0.00239	0.98
	Mean Centering	0.85	0.00077	2.65	0.51	0.00131	1.48	0.49	0.00162	1.45
Polynomial	No Preprocessing	0	0.00203	1.01	0.08	0.00194	1	0	0.00227	1.03
	SNV	0	0.00203	1.01	0.08	0.00194	1	0	0.00227	1.03
	MSC	0	0.00203	1.01	0.08	0.00194	1	0	0.00227	1.03
	**Normalization**	**0.98**	**0.00027**	**7.64**	**0.74**	**0.00095**	**2.03**	**0.81**	**0.00098**	**2.4**
	Moving Average	0	0.00203	1.01	0.08	0.00194	1	0	0.00227	1.03
	Gaussian Filter	0	0.00203	1.01	0.08	0.00194	1	0	0.00227	1.03
	Median Filter	0	0.00203	1.01	0.08	0.00194	1	0	0.00227	1.03
	Detrend	0.02	0.00201	1.02	0.53	0.00231	0.84	0.43	0.00354	0.66
	Mean Centering	0.94	0.00048	4.25	0.69	0.00105	1.84	0.21	0.00915	0.26

Table S2. Results of SVM-R modeling of Khoni cultivars with various spectral preprocessings

Kernel	Preprocessing	Training			Validation			Test		
		R^2	RMSE	RPD	R^2	RMSE	RPD	R^2	RMSE	RPD
Linear	No Preprocessing	**0.99**	**0.00017**	**9.29**	**0.97**	**0.00028**	**6.11**	**0.85**	**0.00065**	**2.68**
	SNV	0.98	0.00019	8.17	0.2	0.00148	1.16	0.45	0.00125	1.39
	MSC	0.98	0.00019	8.25	0.19	0.0015	1.15	0.46	0.00124	1.4
	Normalization	0.99	0.00017	9.53	0.97	0.00031	5.59	0.82	0.00071	2.45
	**Moving Average**	**0.99**	**0.00017**	**9.09**	**0.97**	**0.00028**	**6.1**	**0.85**	**0.00065**	**2.67**
	**Gaussian Filter**	**0.99**	**0.00017**	**9.06**	**0.97**	**0.00028**	**6.13**	**0.85**	**0.00064**	**2.7**
	**Median Filter**	**0.99**	**0.00017**	**9.32**	**0.97**	**0.00029**	**5.93**	**0.85**	**0.00065**	**2.68**
	Detrend	0.99	0.00018	8.83	0.7	0.00091	1.88	0.71	0.0009	1.92
	Mean Centering	0.99	0.00018	9.03	0.97	0.0003	5.74	0.85	0.00065	2.66
RBF	No Preprocessing	0.88	0.00054	2.96	0.4	0.00129	1.33	0.5	0.00119	1.46
	SNV	0.75	0.00078	2.02	0.33	0.00136	1.26	0.37	0.00134	1.3
	MSC	0.74	0.0008	1.97	0.39	0.00129	1.33	0.42	0.00128	1.35
	Normalization	0.89	0.00053	2.99	0.5	0.00117	1.46	0.57	0.0011	1.58
	Moving Average	0.89	0.00052	3.06	0.44	0.00124	1.38	0.54	0.00113	1.53
	Gaussian Filter	0.89	0.00051	3.1	0.44	0.00124	1.39	0.55	0.00113	1.54
	Median Filter	0.91	0.00047	3.36	0.49	0.00119	1.45	0.54	0.00114	1.53
	Detrend	0.89	0.00051	3.07	0.49	0.00119	1.45	0.54	0.00114	1.52
	Mean Centering	0.9	0.0005	3.15	0.44	0.00124	1.38	0.53	0.00115	1.51
Polynomial	No Preprocessing	0	0.00157	1.01	0.01	0.00167	1.03	0.04	0.00172	1.01
	SNV	0.65	0.00093	1.7	0.48	0.0012	1.43	0.44	0.00126	1.37
	MSC	0.03	0.00154	1.03	0.02	0.00164	1.04	0.03	0.00165	1.05
	Normalization	0.92	0.00043	3.66	0.54	0.00456	0.38	0.66	0.00217	0.8
	Moving Average	0	0.00157	1.01	0.01	0.00167	1.03	0.04	0.00172	1.01
	Gaussian Filter	0	0.00157	1.01	0.01	0.00167	1.03	0.04	0.00172	1.01
	Median Filter	0	0.00157	1.01	0.01	0.00167	1.03	0.04	0.00172	1.01
	Detrend	0.99	0.00017	9.07	0.53	0.00113	1.51	0.4	0.0013	1.34
	Mean Centering	0.99	0.00019	8.47	0.11	0.00175	0.98	0.94	0.00234	0.74

Table S3. Results of DT modeling of Khormaei cultivars with various spectral preprocessings

Preprocessing	Max Splits	Training			Validation			Test		
		R^2	RMSE	RPD	R^2	RMSE	RPD	R^2	RMSE	RPD
No Preprocessing	**5**	**0.98**	**0.00026**	**7.74**	**0.99**	**0.00023**	**9.77**	**0.95**	**0.00044**	**4.73**
	**10**	**0.99**	**0.00023**	**8.61**	**0.99**	**0.00019**	**11.57**	**0.96**	**0.00042**	**4.93**
	20	0.99	0.00023	8.61	0.99	0.00019	11.57	0.96	0.00042	4.93
	50	0.99	0.00023	8.61	0.99	0.00019	11.57	0.96	0.00042	4.93
	100	0.99	0.00023	8.61	0.99	0.00019	11.57	0.96	0.00042	4.93
SNV	5	0.86	0.00075	2.65	0.91	0.00296	0.75	0.1	0.0021	0.98
	10	0.9	0.00063	3.14	1.07	0.00308	0.72	0.03	0.00203	1.02
	20	0.9	0.00063	3.14	1.07	0.00308	0.72	0.03	0.00203	1.02
	50	0.9	0.00063	3.14	1.07	0.00308	0.72	0.03	0.00203	1.02
	100	0.9	0.00063	3.14	1.07	0.00308	0.72	0.03	0.00203	1.02
MSC	5	0.9	0.00062	3.24	0.28	0.00243	0.91	0.13	0.00186	1.11
	10	0.95	0.00043	4.69	0.19	0.00234	0.95	0	0.002	1.03
	20	0.95	0.00043	4.69	0.19	0.00234	0.95	0	0.002	1.03
	50	0.95	0.00043	4.69	0.19	0.00234	0.95	0	0.002	1.03
	100	0.95	0.00043	4.69	0.19	0.00234	0.95	0	0.002	1.03
Normalization	5	0.98	0.00026	7.74	0.99	0.00023	9.77	0.95	0.00044	4.73
	10	0.99	0.00023	8.61	0.99	0.00019	11.57	0.96	0.00042	4.93
	20	0.99	0.00023	8.61	0.99	0.00019	11.57	0.96	0.00042	4.93
	50	0.99	0.00023	8.61	0.99	0.00019	11.57	0.96	0.00042	4.93
	100	0.99	0.00023	8.61	0.99	0.00019	11.57	0.96	0.00042	4.93
Moving Average	5	0.98	0.00026	7.74	0.99	0.00023	9.77	0.95	0.00044	4.73
	10	0.99	0.00023	8.61	0.99	0.00019	11.57	0.96	0.00042	4.93
	20	0.99	0.00023	8.61	0.99	0.00019	11.57	0.96	0.00042	4.93
	50	0.99	0.00023	8.61	0.99	0.00019	11.57	0.96	0.00042	4.93
	100	0.99	0.00023	8.61	0.99	0.00019	11.57	0.96	0.00042	4.93
Gaussian Filter	5	0.98	0.00026	7.74	0.99	0.00023	9.77	0.95	0.00044	4.73
	10	0.99	0.00023	8.61	0.99	0.00019	11.57	0.96	0.00042	4.93
	20	0.99	0.00023	8.61	0.99	0.00019	11.57	0.96	0.00042	4.93
	50	0.99	0.00023	8.61	0.99	0.00019	11.57	0.96	0.00042	4.93
	100	0.99	0.00023	8.61	0.99	0.00019	11.57	0.96	0.00042	4.93
Median Filter	5	0.98	0.00026	7.74	0.99	0.00023	9.77	0.95	0.00044	4.73
	10	0.99	0.00023	8.61	0.99	0.00019	11.57	0.96	0.00042	4.93
	20	0.99	0.00023	8.61	0.99	0.00019	11.57	0.96	0.00042	4.93
	50	0.99	0.00023	8.61	0.99	0.00019	11.57	0.96	0.00042	4.93
	100	0.99	0.00023	8.61	0.99	0.00019	11.57	0.96	0.00042	4.93
Detrend	5	0.79	0.00091	2.19	0.37	0.00251	0.88	0.37	0.00234	0.88
	10	0.93	0.0005	3.96	0.6	0.00271	0.82	0.64	0.00256	0.81

	20	0.93	0.0005	3.96	0.6	0.00271	0.82	0.64	0.00256	0.81
	50	0.93	0.0005	3.96	0.6	0.00271	0.82	0.64	0.00256	0.81
	100	0.93	0.0005	3.96	0.6	0.00271	0.82	0.64	0.00256	0.81
Mean Centering	5	0.98	0.00026	7.74	0.99	0.00023	9.77	0.95	0.00044	4.73
	10	0.99	0.00023	8.61	0.99	0.00019	11.57	0.96	0.00042	4.93
	20	0.99	0.00023	8.61	0.99	0.00019	11.57	0.96	0.00042	4.93
	50	0.99	0.00023	8.61	0.99	0.00019	11.57	0.96	0.00042	4.93
	100	0.99	0.00023	8.61	0.99	0.00019	11.57	0.96	0.00042	4.93

Table S4. Results of DT modeling of Khoni cultivars with various spectral preprocessings

Preprocessing	Max Splits	Training			Validation			Test		
		R^2	RMSE	RPD	R^2	RMSE	RPD	R^2	RMSE	RPD
No Preprocessing	**5**	**0.97**	**0.00028**	**6.36**	**0.94**	**0.00031**	**4.31**	**0.94**	**0.00034**	**4.21**
	**10**	**0.99**	**0.00018**	**9.77**	**0.97**	**0.00022**	**6.08**	**0.96**	**0.00026**	**5.41**
	20	0.99	0.00018	9.77	0.97	0.00022	6.08	0.96	0.00026	5.41

	50	0.99	0.00018	9.77	0.97	0.00022	6.08	0.96	0.00026	5.41
	100	0.99	0.00018	9.77	0.97	0.00022	6.08	0.96	0.00026	5.41
SNV	5	0.88	0.00061	2.9	0.73	0.00169	0.79	0.38	0.00108	1.31
	10	0.94	0.00043	4.11	0.79	0.00172	0.77	0.5	0.00097	1.46
	20	0.94	0.00043	4.11	0.79	0.00172	0.77	0.5	0.00097	1.46
	50	0.94	0.00043	4.11	0.79	0.00172	0.77	0.5	0.00097	1.46
	100	0.94	0.00043	4.11	0.79	0.00172	0.77	0.5	0.00097	1.46
MSC	5	0.85	0.00066	2.64	0.79	0.00172	0.77	0.46	0.001	1.41
	10	0.96	0.00033	5.31	0.56	0.00161	0.83	0.51	0.00096	1.48
	20	0.96	0.00033	5.31	0.56	0.00161	0.83	0.51	0.00096	1.48
	50	0.96	0.00033	5.31	0.56	0.00161	0.83	0.51	0.00096	1.48
	100	0.96	0.00033	5.31	0.56	0.00161	0.83	0.51	0.00096	1.48
Normalization	**5**	**0.97**	**0.00028**	**6.36**	**0.94**	**0.00031**	**4.31**	**0.94**	**0.00034**	**4.21**
	**10**	**0.99**	**0.00018**	**9.77**	**0.97**	**0.00022**	**6.08**	**0.96**	**0.00026**	**5.41**
	20	0.99	0.00018	9.77	0.97	0.00022	6.08	0.96	0.00026	5.41
	50	0.99	0.00018	9.77	0.97	0.00022	6.08	0.96	0.00026	5.41
	100	0.99	0.00018	9.77	0.97	0.00022	6.08	0.96	0.00026	5.41
Moving Average	**5**	**0.97**	**0.00028**	**6.36**	**0.94**	**0.00031**	**4.31**	**0.94**	**0.00034**	**4.21**
	**10**	**0.99**	**0.00018**	**9.77**	**0.97**	**0.00022**	**6.08**	**0.96**	**0.00026**	**5.41**
	20	0.99	0.00018	9.77	0.97	0.00022	6.08	0.96	0.00026	5.41
	50	0.99	0.00018	9.77	0.97	0.00022	6.08	0.96	0.00026	5.41
	100	0.99	0.00018	9.77	0.97	0.00022	6.08	0.96	0.00026	5.41
Gaussian Filter	**5**	**0.97**	**0.00028**	**6.36**	**0.94**	**0.00031**	**4.31**	**0.94**	**0.00034**	**4.21**
	**10**	**0.99**	**0.00018**	**9.77**	**0.97**	**0.00022**	**6.08**	**0.96**	**0.00026**	**5.41**
	20	0.99	0.00018	9.77	0.97	0.00022	6.08	0.96	0.00026	5.41
	50	0.99	0.00018	9.77	0.97	0.00022	6.08	0.96	0.00026	5.41
	100	0.99	0.00018	9.77	0.97	0.00022	6.08	0.96	0.00026	5.41
Median Filter	**5**	**0.97**	**0.00028**	**6.36**	**0.94**	**0.00031**	**4.31**	**0.94**	**0.00034**	**4.21**
	**10**	**0.99**	**0.00018**	**9.77**	**0.97**	**0.00022**	**6.08**	**0.96**	**0.00026**	**5.41**
	20	0.99	0.00018	9.77	0.97	0.00022	6.08	0.96	0.00026	5.41
	50	0.99	0.00018	9.77	0.97	0.00022	6.08	0.96	0.00026	5.41
	100	0.99	0.00018	9.77	0.97	0.00022	6.08	0.96	0.00026	5.41
Detrend	5	0.92	0.0005	3.51	0.31	0.00107	1.24	0.46	0.00101	1.4
	10	0.97	0.00031	5.58	0.37	0.00103	1.3	0.45	0.00102	1.39
	20	0.97	0.00031	5.58	0.37	0.00103	1.3	0.45	0.00102	1.39
	50	0.97	0.00031	5.58	0.37	0.00103	1.3	0.45	0.00102	1.39
	100	0.97	0.00031	5.58	0.37	0.00103	1.3	0.45	0.00102	1.39
Mean Centering	**5**	**0.97**	**0.00028**	**6.36**	**0.94**	**0.00031**	**4.31**	**0.94**	**0.00034**	**4.21**

**10**	**0.99**	**0.00018**	**9.77**	**0.97**	**0.00022**	**6.08**	**0.96**	**0.00026**	**5.41**
20	0.99	0.00018	9.77	0.97	0.00022	6.08	0.96	0.00026	5.41
50	0.99	0.00018	9.77	0.97	0.00022	6.08	0.96	0.00026	5.41
100	0.99	0.00018	9.77	0.97	0.00022	6.08	0.96	0.00026	5.41

Table S5. Results of PLSR modeling of Khormaei and Khoni cultivars with various spectral preprocessings

**Kernel**	**Preprocessing**	**Component**	**Training**			**Validation**			**Test**		
			**R^2**	**RMSE**	**RPD**	**R^2**	**RMSE**	**RPD**	**R^2**	**RMSE**	**RPD**
Khormaei	**No Preprocessing**	5	0.99	0.000121	16.61	0.53	0.001372	1.51	0.57	0.001489	1.57
	SNV	5	0.59	0.001367	1.59	0.59	0.001203	1.61	0	0.001846	0.96
	MSC	5	0.80	0.000908	2.28	0.43	0.001686	1.37	0.21	0.001596	1.16

	Normalization	5	0.75	0.000989	2.01	0.78	0.00094	2.21	0.67	0.00122	1.81
	Moving Average	5	0.76	0.001031	2.07	0.69	0.001071	1.85	0.78	0.000942	2.22
	Gaussian Filter	5	0.73	0.000983	1.96	0.67	0.001247	1.79	0.76	0.001147	2.09
	Median Filter	5	0.76	0.001067	2.06	0.71	0.000985	1.91	0.73	0.00095	1.98
	Detrend	5	0.96	0.000404	5.03	0.34	0.001702	1.27	0.24	0.001826	1.18
	**Mean Centering**	5	0.99	0.000176	11.1	0.77	0.001054	2.17	0.43	0.001574	1.36
	**No Preprocessing**	**8**	**0.98**	**0.000207**	**7.55**	**0.97**	**0.000284**	**6.33**	**0.82**	**0.000713**	**2.42**
	SNV	8	0.94	0.000379	4.12	0.42	0.001326	1.36	0.35	0.001673	1.03
	MSC	8	0.94	0.000383	4.07	0.42	0.001323	1.36	0.31	0.001681	1.03
	**Normalization**	**8**	**0.98**	**0.000225**	**6.92**	**0.96**	**0.000331**	**5.44**	**0.86**	**0.000615**	**2.81**
Khoni	Moving Average	8	0.98	0.00022	7.08	0.97	0.000279	6.44	0.81	0.000723	2.39
	Gaussian Filter	8	0.98	0.000228	6.84	0.97	0.000281	6.4	0.81	0.000736	2.34
	Median Filter	8	0.98	0.000215	7.25	0.98	0.000269	6.67	0.82	0.000714	2.42
	Detrend	8	0.98	0.000205	7.6	0.81	0.000757	2.37	0.49	0.001192	1.45
	Mean Centering	8	0.98	0.000207	7.55	0.97	0.000284	6.33	0.82	0.000713	2.42

Table S6. Results of SVM-R modeling of Khormaei cultivars with various spectral preprocessings

**Kernel**	**Preprocessing**	**Training**			**Validation**			**Test**		
		**R^2**	**RMSE**	**RPD**	**R^2**	**RMSE**	**RPD**	**R^2**	**RMSE**	**RPD**
	No Preprocessing	0.98	0.0003	7.05	0.85	0.00078	2.7	0.24	0.00177	0.93
Linear	SNV	0.98	0.00032	6.68	0.37	0.00162	1.3	0.59	0.00201	0.82
	MSC	0.98	0.00031	6.77	0.37	0.00161	1.31	0.65	0.00205	0.8

	Normalization	0.98	0.0003	6.99	0.79	0.00092	2.28	0.21	0.00175	0.94
	Moving Average	0.98	0.0003	6.94	0.83	0.00085	2.49	0.41	0.00189	0.87
	Gaussian Filter	0.98	0.00031	6.89	0.82	0.00087	2.43	0.52	0.00196	0.84
	Median Filter	0.98	0.0003	7.02	0.83	0.00084	2.52	0.27	0.0018	0.91
	Detrend	0.98	0.00032	6.57	0.51	0.00143	1.48	0.91	0.0022	0.75
	Mean Centering	0.98	0.0003	7.04	0.86	0.00077	2.73	0.24	0.00177	0.93
RBF	**No Preprocessing**	**0.95**	**0.00044**	**4.74**	**0.5**	**0.00144**	**1.46**	**0.71**	**0.00085**	**1.93**
	SNV	0.65	0.00123	1.71	0.05	0.0021	1.01	0.34	0.00184	0.89
	MSC	0.67	0.00119	1.77	0.06	0.0021	1	0.39	0.00188	0.88
	Normalization	0.89	0.0007	2.99	0.35	0.00164	1.28	0.43	0.0012	1.37
	Moving Average	0.86	0.00078	2.72	0.35	0.00165	1.28	0.54	0.00109	1.52
	Gaussian Filter	0.86	0.00078	2.7	0.34	0.00165	1.27	0.56	0.00106	1.55
	Median Filter	0.88	0.00073	2.9	0.34	0.00166	1.27	0.43	0.0012	1.37
	Detrend	0.64	0.00125	1.69	0.14	0.00218	0.97	0.24	0.00177	0.93
	Mean Centering	0.86	0.00078	2.71	0.36	0.00164	1.29	0.56	0.00106	1.56
Polynomial	No Preprocessing	0	0.00208	1.01	0.02	0.00206	1.02	0.64	0.00204	0.81
	SNV	0.42	0.00249	0.85	0.2	0.00182	1.16	0.64	0.00343	0.48
	MSC	0	0.00208	1.01	0.02	0.00206	1.02	0.64	0.00204	0.81
	**Normalization**	**0.29**	**0.00523**	**0.4**	**0.45**	**0.00246**	**0.86**	**0.85**	**0.00062**	**2.65**
	Moving Average	0	0.00208	1.01	0.02	0.00206	1.02	0.64	0.00204	0.81
	Gaussian Filter	0	0.00208	1.01	0.02	0.00206	1.02	0.64	0.00204	0.81
	Median Filter	0	0.00208	1.01	0.02	0.00206	1.02	0.64	0.00204	0.81
	Detrend	0.2	0.00597	0.35	0.04	0.00765	0.28	0.24	0.00693	0.24
	**Mean Centering**	**0.87**	**0.00076**	**2.76**	**0.72**	**0.00336**	**0.63**	**0.8**	**0.00071**	**2.33**

Table S7. Results of SVM-R modeling of Khoni cultivars with various spectral preprocessings

Kernel	Preprocessing	Training			Validation			Test		
		R^2	RMSE	RPD	R^2	RMSE	RPD	R^2	RMSE	RPD
Linear	**No Preprocessing**	**0.98**	**0.0002**	**7.99**	**0.93**	**0.00042**	**3.86**	**0.86**	**0.00059**	**2.78**
	SNV	0.98	0.0002	7.77	0.38	0.00182	0.88	0.35	0.00128	1.28
	MSC	0.98	0.0002	7.8	0.36	0.00181	0.89	0.31	0.00132	1.24

	**Normalization**	**0.98**	**0.0002**	**7.89**	**0.92**	**0.00045**	**3.56**	**0.88**	**0.00055**	**2.98**
	Moving Average	0.98	0.0002	8.06	0.92	0.00043	3.74	0.85	0.00061	2.7
	Gaussian Filter	0.98	0.00019	8.16	0.92	0.00044	3.66	0.86	0.0006	2.72
	**Median Filter**	**0.98**	**0.0002**	**8.14**	**0.93**	**0.00041**	**3.92**	**0.87**	**0.00058**	**2.84**
	Detrend	0.98	0.00021	7.7	0.86	0.00058	2.78	0.65	0.00093	1.76
	**Mean Centering**	**0.98**	**0.0002**	**8.1**	**0.93**	**0.00041**	**3.94**	**0.86**	**0.00059**	**2.8**
RBF	No Preprocessing	0.89	0.00053	2.99	0.37	0.00123	1.3	0.37	0.00126	1.3
	SNV	0.81	0.00068	2.33	0.08	0.00149	1.08	0.38	0.00125	1.31
	MSC	0.81	0.00068	2.33	0.08	0.00149	1.08	0.38	0.00125	1.31
	Normalization	0.87	0.00056	2.83	0.34	0.00126	1.27	0.32	0.00131	1.25
	Moving Average	0.89	0.00052	3.03	0.38	0.00122	1.32	0.38	0.00125	1.31
	Gaussian Filter	0.88	0.00053	2.98	0.37	0.00123	1.3	0.37	0.00126	1.3
	Median Filter	0.9	0.00051	3.13	0.42	0.00118	1.35	0.41	0.00122	1.34
	Detrend	0.82	0.00067	2.37	0.44	0.00115	1.39	0.44	0.00119	1.38
	Mean Centering	0.9	0.00051	3.14	0.42	0.00118	1.36	0.41	0.00122	1.34
Polynomial	No Preprocessing	0	0.00157	1.01	0.38	0.00182	0.88	0	0.00159	1.03
	SNV	0.35	0.00241	0.66	0.81	0.00209	0.77	0.34	0.0029	0.57
	MSC	0	0.00157	1.01	0.38	0.00182	0.88	0	0.00159	1.03
	Normalization	0.61	0.00442	0.36	0.38	0.00122	1.31	0.39	0.00187	0.88
	Moving Average	0	0.00157	1.01	0.38	0.00182	0.88	0	0.00159	1.03
	Gaussian Filter	0	0.00157	1.01	0.38	0.00182	0.88	0	0.00159	1.03
	Median Filter	0	0.00157	1.01	0.38	0.00182	0.88	0	0.00159	1.03
	Detrend	0.58	0.01165	0.14	0.32	0.00681	0.23	0.54	0.01369	0.12
	Mean Centering	0.99	0.00019	8.55	0.77	0.00075	2.15	0.31	0.00132	1.24

Table S8. Results of DT modeling of Khormaei cultivars with various spectral preprocessings

**Preprocessing**	**Max Splits**	**Training**			**Validation**			**Test**		
		**R^2**	**RMSE**	**RPD**	**R^2**	**RMSE**	**RPD**	**R^2**	**RMSE**	**RPD**
No Preprocessing	**5**	**0.99**	**0.00026**	**8.54**	**0.99**	**0.00022**	**9.1**	**0.9**	**0.00056**	**3.22**
	**10**	**0.99**	**0.00024**	**9.4**	**0.99**	**0.00021**	**9.58**	**0.9**	**0.00054**	**3.32**
	20	0.99	0.00024	9.4	0.99	0.00021	9.58	0.9	0.00054	3.32

	50	0.99	0.00024	9.4	0.99	0.00021	9.58	0.9	0.00054	3.32
	100	0.99	0.00024	9.4	0.99	0.00021	9.58	0.9	0.00054	3.32
SNV	5	0.81	0.00095	2.32	1.36	0.00298	0.67	0.06	0.00179	1
	10	0.87	0.0008	2.76	1.43	0.00303	0.66	0.23	0.00193	0.93
	20	0.87	0.0008	2.76	1.43	0.00303	0.66	0.23	0.00193	0.93
	50	0.87	0.0008	2.76	1.43	0.00303	0.66	0.23	0.00193	0.93
	100	0.87	0.0008	2.76	1.43	0.00303	0.66	0.23	0.00193	0.93
MSC	5	0.88	0.00078	2.86	1.55	0.0031	0.65	0.89	0.00239	0.75
	10	0.93	0.00059	3.74	1.44	0.00303	0.66	1.05	0.00249	0.72
	20	0.93	0.00059	3.74	1.44	0.00303	0.66	1.05	0.00249	0.72
	50	0.93	0.00059	3.74	1.44	0.00303	0.66	1.05	0.00249	0.72
	100	0.93	0.00059	3.74	1.44	0.00303	0.66	1.05	0.00249	0.72
Normalization	**5**	**0.99**	**0.00026**	**8.54**	**0.99**	**0.00022**	**9.1**	**0.9**	**0.00056**	**3.22**
	**10**	**0.99**	**0.00024**	**9.4**	**0.99**	**0.00021**	**9.58**	**0.9**	**0.00054**	**3.32**
	20	0.99	0.00024	9.4	0.99	0.00021	9.58	0.9	0.00054	3.32
	50	0.99	0.00024	9.4	0.99	0.00021	9.58	0.9	0.00054	3.32
	100	0.99	0.00024	9.4	0.99	0.00021	9.58	0.9	0.00054	3.32
Moving Average	**5**	**0.99**	**0.00026**	**8.54**	**0.99**	**0.00022**	**9.1**	**0.9**	**0.00056**	**3.22**
	**10**	**0.99**	**0.00024**	**9.4**	**0.99**	**0.00021**	**9.58**	**0.9**	**0.00054**	**3.32**
	20	0.99	0.00024	9.4	0.99	0.00021	9.58	0.9	0.00054	3.32
	50	0.99	0.00024	9.4	0.99	0.00021	9.58	0.9	0.00054	3.32
	100	0.99	0.00024	9.4	0.99	0.00021	9.58	0.9	0.00054	3.32
Gaussian Filter	**5**	**0.99**	**0.00026**	**8.54**	**0.99**	**0.00022**	**9.1**	**0.9**	**0.00056**	**3.22**
	**10**	**0.99**	**0.00024**	**9.4**	**0.99**	**0.00023**	**8.91**	**0.9**	**0.00054**	**3.32**
	20	0.99	0.00024	9.4	0.99	0.00023	8.91	0.9	0.00054	3.32
	50	0.99	0.00024	9.4	0.99	0.00023	8.91	0.9	0.00054	3.32
	100	0.99	0.00024	9.4	0.99	0.00023	8.91	0.9	0.00054	3.32
Median Filter	**5**	**0.99**	**0.00026**	**8.54**	**0.99**	**0.00022**	**9.1**	**0.9**	**0.00056**	**3.22**
	**10**	**0.99**	**0.00024**	**9.4**	**0.99**	**0.00021**	**9.58**	**0.9**	**0.00054**	**3.32**
	20	0.99	0.00024	9.4	0.99	0.00021	9.58	0.9	0.00054	3.32
	50	0.99	0.00024	9.4	0.99	0.00021	9.58	0.9	0.00054	3.32
	100	0.99	0.00024	9.4	0.99	0.00021	9.58	0.9	0.00054	3.32
Detrend	5	0.88	0.00076	2.9	0.95	0.00271	0.74	0.61	0.0022	0.82
	10	0.9	0.00068	3.27	0.9	0.00267	0.75	0.63	0.00222	0.81
	20	0.9	0.00068	3.27	0.9	0.00267	0.75	0.63	0.00222	0.81
	50	0.9	0.00068	3.27	0.9	0.00267	0.75	0.63	0.00222	0.81
	100	0.9	0.00068	3.27	0.9	0.00267	0.75	0.63	0.00222	0.81
Mean Centering	**5**	**0.99**	**0.00026**	**8.54**	**0.99**	**0.00022**	**9.1**	**0.9**	**0.00056**	**3.22**

	**10**	**0.99**	**0.00024**	**9.4**	**0.99**	**0.00021**	**9.58**	**0.9**	**0.00054**	**3.32**
	20	0.99	0.00024	9.4	0.99	0.00021	9.58	0.9	0.00054	3.32
	50	0.99	0.00024	9.4	0.99	0.00021	9.58	0.9	0.00054	3.32
	100	0.99	0.00024	9.4	0.99	0.00021	9.58	0.9	0.00054	3.32

Table S9. Results of DT modeling of Khoni cultivars with various spectral preprocessings

**Preprocessing**	**Max Splits**	**Training**			**Validation**			**Test**		
		**R^2**	**RMSE**	**RPD**	**R^2**	**RMSE**	**RPD**	**R^2**	**RMSE**	**RPD**
No Preprocessing	**5**	**0.97**	**0.00029**	**5.5**	**0.94**	**0.00038**	**4.3**	**0.94**	**0.00038**	**4.38**
	**10**	**0.98**	**0.00023**	**7**	**0.97**	**0.00026**	**6.37**	**0.97**	**0.0003**	**5.69**

	20	0.98	0.00023	7	0.97	0.00026	6.37	0.97	0.0003	5.69
	50	0.98	0.00023	7	0.97	0.00026	6.37	0.97	0.0003	5.69
	100	0.98	0.00023	7	0.97	0.00026	6.37	0.97	0.0003	5.69
SNV	5	0.87	0.00058	2.79	0.36	0.00126	1.29	0.35	0.00131	1.28
	10	0.92	0.00044	3.62	0.37	0.00125	1.3	0.26	0.0014	1.2
	20	0.92	0.00044	3.62	0.37	0.00125	1.3	0.26	0.0014	1.2
	50	0.92	0.00044	3.62	0.37	0.00125	1.3	0.26	0.0014	1.2
	100	0.92	0.00044	3.62	0.37	0.00125	1.3	0.26	0.0014	1.2
MSC	5	0.87	0.00058	2.79	0.44	0.00118	1.38	0.44	0.00122	1.38
	10	0.92	0.00044	3.62	0.41	0.00121	1.35	0.43	0.00123	1.37
	20	0.92	0.00044	3.62	0.41	0.00121	1.35	0.43	0.00123	1.37
	50	0.92	0.00044	3.62	0.41	0.00121	1.35	0.43	0.00123	1.37
	100	0.92	0.00044	3.62	0.41	0.00121	1.35	0.43	0.00123	1.37
Normalization	**5**	**0.97**	**0.00029**	**5.5**	**0.94**	**0.00038**	**4.3**	**0.94**	**0.00038**	**4.38**
	**10**	**0.98**	**0.00023**	**7**	**0.97**	**0.00026**	**6.37**	**0.97**	**0.0003**	**5.69**
	20	0.98	0.00023	7	0.97	0.00026	6.37	0.97	0.0003	5.69
	50	0.98	0.00023	7	0.97	0.00026	6.37	0.97	0.0003	5.69
	100	0.98	0.00023	7	0.97	0.00026	6.37	0.97	0.0003	5.69
Moving Average	**5**	**0.97**	**0.00029**	**5.5**	**0.94**	**0.00038**	**4.3**	**0.94**	**0.00038**	**4.38**
	**10**	**0.98**	**0.00023**	**7**	**0.97**	**0.00026**	**6.37**	**0.97**	**0.0003**	**5.69**
	20	0.98	0.00023	7	0.97	0.00026	6.37	0.97	0.0003	5.69
	50	0.98	0.00023	7	0.97	0.00026	6.37	0.97	0.0003	5.69
	100	0.98	0.00023	7	0.97	0.00026	6.37	0.97	0.0003	5.69
Gaussian Filter	**5**	**0.97**	**0.00029**	**5.5**	**0.94**	**0.00038**	**4.3**	**0.94**	**0.00038**	**4.38**
	**10**	**0.98**	**0.00023**	**7**	**0.97**	**0.00026**	**6.37**	**0.97**	**0.0003**	**5.69**
	20	0.98	0.00023	7	0.97	0.00026	6.37	0.97	0.0003	5.69
	50	0.98	0.00023	7	0.97	0.00026	6.37	0.97	0.0003	5.69
	100	0.98	0.00023	7	0.97	0.00026	6.37	0.97	0.0003	5.69
Median Filter	**5**	**0.97**	**0.00029**	**5.5**	**0.94**	**0.00038**	**4.3**	**0.94**	**0.00038**	**4.38**
	**10**	**0.98**	**0.00023**	**7**	**0.97**	**0.00026**	**6.37**	**0.97**	**0.0003**	**5.69**
	20	0.98	0.00023	7	0.97	0.00026	6.37	0.97	0.0003	5.69
	50	0.98	0.00023	7	0.97	0.00026	6.37	0.97	0.0003	5.69
	100	0.98	0.00023	7	0.97	0.00026	6.37	0.97	0.0003	5.69
Detrend	5	0.9	0.00051	3.18	0.55	0.00105	1.55	0.65	0.00096	1.75
	10	0.95	0.00036	4.45	0.56	0.00105	1.55	0.7	0.0009	1.88
	20	0.95	0.00036	4.45	0.56	0.00105	1.55	0.7	0.0009	1.88
	50	0.95	0.00036	4.45	0.56	0.00105	1.55	0.7	0.0009	1.88
	100	0.95	0.00036	4.45	0.56	0.00105	1.55	0.7	0.0009	1.88

	**5**	**0.97**	**0.00029**	**5.5**	**0.94**	**0.00038**	**4.3**	**0.94**	**0.00038**	**4.38**
	**10**	**0.98**	**0.00023**	**7**	**0.97**	**0.00026**	**6.37**	**0.97**	**0.0003**	**5.69**
Mean Centering	20	0.98	0.00023	7	0.97	0.00026	6.37	0.97	0.0003	5.69
	50	0.98	0.00023	7	0.97	0.00026	6.37	0.97	0.0003	5.69
	100	0.98	0.00023	7	0.97	0.00026	6.37	0.97	0.0003	5.69

Table S10. Results of PLSR modeling of Khormaei and Khoni cultivars with various spectral preprocessings

Kernel	Preprocessing	Component	Training			Validation			Test		
			R^2	RMSE	RPD	R^2	RMSE	RPD	R^2	RMSE	RPD
Khormaei	**No Preprocessing**	**9**	**0.95**	**0.000449**	**4.65**	**0.79**	**0.00092**	**2.23**	**0.44**	**307**	**0.96**

	SNV	9	0.81	0.000904	2.31	0.22	0.00219	0.94	0.35	295	1
	MSC	9	0.66	0.001202	1.74	0.18	0.005489	0.37	0.35	295	1
	Normalization	9	0.72	0.001102	1.89	0.37	0.001575	1.3	0.37	298	0.99
	Moving Average	9	0.94	0.000513	4.07	0.75	0.000987	2.08	0.44	308	0.96
	Gaussian Filter	9	0.93	0.00055	3.8	0.71	0.001077	1.9	0.44	308	0.96
	**Median Filter**	**9**	**0.97**	**0.000345**	**6.06**	**0.75**	**0.000992**	**2.07**	**0.42**	**305**	**0.97**
	Detrend	9	0.96	0.000401	5.21	0.19	0.001786	1.15	0.35	295	1
	Mean Centering	9	0.95	0.000449	4.65	0.79	0.00092	2.23	0.43	307	0.96
Khoni	**No Preprocessing**	**8**	**0.63**	**0.000953**	**1.66**	**0.79**	**0.000883**	**2.27**	**0.38**	**0.001277**	**1.05**
	SNV	8	0.42	0.001199	1.32	0.18	0.001761	1.14	0.38	0.001278	1.05
	MSC	8	0.4	0.001218	1.3	0.1	0.001842	1.09	0.36	0.001309	1.03
	Normalization	8	0.63	0.000953	1.66	0.79	0.000891	2.25	0.39	0.001275	1.05
	Moving Average	8	0.63	0.000958	1.65	0.79	0.000886	2.26	0.36	0.001302	1.03
	Gaussian Filter	8	0.62	0.000972	1.63	0.74	0.000982	2.04	0.45	0.001361	0.99
	**Median Filter**	**8**	**0.72**	**0.000826**	**1.92**	**0.35**	**0.00157**	**1.28**	**0.44**	**0.00124**	**1.08**
	Detrend	8	0.46	0.001157	1.37	0.23	0.001702	1.18	0.38	0.001276	1.05
	Mean Centering	8	0.63	0.000953	1.66	0.79	0.000883	2.27	0.38	0.001277	1.05
